# Ozonized Gel Against Four *Candida* Species: A Pilot Study and Clinical Perspectives

**DOI:** 10.3390/ma13071731

**Published:** 2020-04-08

**Authors:** Vincenzina Monzillo, Fabiola Lallitto, Alba Russo, Claudio Poggio, Andrea Scribante, Carla Renata Arciola, Francesco Rocco Bertuccio, Marco Colombo

**Affiliations:** 1 Microbiology and Virology Unit IRCCS, 27100 Pavia, Italy; vincenzina.monzillo@unipv.it (V.M.); fabiola.lallitto01@universitadipavia.it (F.L.); 2 Infectious Diseases Unit, Internal Medicine and Medical Therapy Department, University of Pavia, 27100 Pavia, Italy; 3 Department of Clinical-Surgical, Diagnostic and Pediatric Sciences, Section of Dentistry, University of Pavia, 27100 Pavia, Italy; alba.russo01@universitadipavia.it (A.R.); marco.colombo@unipv.it (M.C.); 4 Laboratorio di Patologia delle Infezioni Associate all’Impianto, IRCCS Istituto Ortopedico Rizzoli, via di Barbiano 1/10, 40136 Bologna, Italy; 5 Department of Experimental, Diagnostic and Specialty Medicine, University of Bologna, via San Giacomo 14, 40126 Bologna, Italy; 6 Respiratory Diseases Department, Policlinico San Matteo, 27100 Pavia, Italy; francesco.bertuccio01@gmail.com

**Keywords:** ozone, ozonized olive oil, antimycotic activity, *Candida*

## Abstract

Ozone therapy can display a wide range of clinical beneficial effects, including antimicrobial, immune-stimulant, analgesic, anti-hypoxic actions. However, there is still a paucity of data regarding the ozone fungicide activity. Oral *Candida* is the most common fungal infection in the mouth among denture wearers and people with weakened immune systems. In the case of generalized candidiasis or immunocompromised patients, systemic therapy is needed, while localized infections are treated with topic medications. However, many *Candida* strains are resistant to antifungal drugs. The aim of this preliminary analysis is to evaluate the antimycotic efficacy of a new ozonided oil (GeliO_3_), as a possible terapeutic alternative in local treatments of these infections, compared to chlorhexidine digluconate (Plak gel®). Chlorhexidine is a chemical synthesis disinfectant with a broad-spectrum antiseptic action, active against bacteria and fungi. Antimycotic activity was tested against the following four *Candida* species: *C. albicans*, *C. parapsilosis*, *C. glabrata*, *C. tropicalis*, through an agar diffusion method. No significant differences were found between the growth inhibition zone diameters of the ozonized gel and chlorhexidine. The results indicated that the ozonized gel may help to combat *Candida* infections. Moreover, useful applications could be used to counteract *Candida* colonization of endosseous implants.

## 1. Introduction

Candidiasis is one of the most common human opportunistic fungal infections, caused by a genus of yeasts called *Candida* [1]. *Candida* is located in the oral cavity and on mucosal surfaces, especially those of the gastrointestinal tract [2]. Many species are commensal symbiotic microorganisms. However, when the integrity of the mucosal barriers is compromised or the immune system is weakened, they can invade tissues and cause diseases [3]. The incidence of fungal infections has been increasing in recent decades [4]. The expression of *Candida* virulence in the oral cavity is strongly correlated with some predisposing factors, such as the use of dentures, hypo-salivation, prolonged therapy with antibiotics or immunosuppressive drugs, local trauma, malnutrition, endocrine disorders, and people’s increased longevity, all elements that determine an impairment of the immune system [5]. Usually, the treatment of these fungal infections is based on polienes and imidazol derivative drugs [6]. Short-term treatment has demonstrated an increase in the fungal resistance to these medicaments; the high morbidity rate associated with resistant mycosis indicates that the search for new alternative treatments is very important [7]. Regarding systemic candidiasis, echinocandins represent an adequate alternative solution while, for localized oral infections, chlorhexidine is often prescribed by dentists as an antiseptic mouthwash and a denture disinfectant despite its cytotoxicity [8,9]. Previous studies demonstrated an in vitro antibacterial activity for ozonized vegetable oils against microorganisms [10,11]. Ozone (O_3_) is a highly reactive molecule composed of three oxygen atoms that acts as both an oxidant and oxidizer [12]. The reliable microbiologic and metabolic properties of ozone make it a useful disinfectant with a wide range of activities [13,14]. Ozone demonstrated its antimicrobial effect on bacteria, virus, protozoa and fungi, besides its immunomodulatory, anti-hypoxic, biosynthetic, and anti-inflammatory properties [15,16]. When bacteria are exposed to ozone in vitro, the phospholipids and lipoproteins of the bacterial cell envelope are oxidized. That mechanism disrupts the cytosolic membrane integrity, leading ozone to infiltrate the microorganisms and oxidize glycoproteins and glycolipids, blocking enzymatic function. Moreover, evidence has demonstrated that ozone interacts with fungal cell walls like bacteria [17,18].

In addition, in cell culture assays, it did not show a cytotoxic effect on fibroblasts or keratinocytes and it induced fibroblast migration, which could aid the wound-healing process [19]. 

Ozone therapy have been experimented with for various aspects of dentistry. To date, it has shown efficacy in managing wound healing, dental caries, oral lichen planus, gingivitis and periodontitis, halitosis, osteonecrosis of the jaw, post-surgical pain, plaque and biofilms, root canal treatment, dentin hypersensitivity, temporomandibular joint disorders, and teeth whitening [20]. 

Moreover, the various beneficial effects of ozone therapy could be applied in both dental and orthopedic implantology. Modern strategies to prevent implant-associated infections are geared towards treatments and technologies capable of preventing/resisting infections while promoting repair and osseous integration [21,22,23,24].The promotion of peri-implant bone-healing of endosseous implants by an ozonized oil has been experimentally studied in immunosuppressed rabbits [25]. A better osseointegration process in endosseous implants treated with photobiomodulation and ozone therapy has been described [26]. Modifications have been proposed to improve their integration but also to confer anti-infective properties [27,28,29]. Ozone-ultraviolet treatment of orthopedic titanium implants improved bone-implant osseointegration. Osteoblasts cultured on ozone-ultraviolet-treated surfaces displayed a significantly higher expression of osteoblast markers, like bone morphogenetic protein 2, and osteocalcin [30]. Ozonized oils and other ozone treatments exhibited the capability to promote the bone integration of dental and orthopedic implants while expressing antimicrobial and even immunomodulatory effects [25,26,31]. However, the fungicidal potential of ozone therapy is still poorly explored. The purpose of this pilot study is to evaluate the antimycotic activity of a new ozonized gel. The ozonized gel is obtained from the chemical reaction between ozone and unsaturated fatty acids of vegetable oils. The effectiveness of the ozonized gel (GeliO_3_) was compared to 0.2% chlorhexidine digluconate (Plak gel®) through an agar diffusion assay. Each treatment was tested against the four species most commonly associated with candidiasis: Candida albicans (65.3%), Candida glabrata (11.3%), Candida tropicalis (7.2%) and C. parapsilosis (6.0%). C. albicans remains the most commonly isolated, but the proportion relative to other Candida species has decreased over time (from 71 to 65%). This was accompanied by an increasing incidence of C. glabrata, C. tropicalis, and C. parapsilosis [32].

## 2. Materials and Methods 

The samples of ozonized gel (GeliO_3_), composed of Bio-ozonized olive oil and a synthetic amorphous silica gel with a peroxide index of 20 mEq O_2_/Kg, were supplied by Bioemmei Srl, Vicenza, Italy. 

Chlorhexidine digluconate in a gel containing polyoxymethyleneand hydrogenated ricinus oil (Plak gel® 0,2%) was provided by Polifarma Benessere s.r.l., Roma (RM), Italy.

Candida strains were randomly selected from the old stocks of the Microbiology and Virology laboratory, IRCCS Policlinico San Matteo, Pavia, Italy

A total of 12 Candida strains, belonging to the four species most implicated in human infection [32], were tested in this study: C. albicans (n = 3), C. parapsilosis (n = 3), C. glabrata (n = 3), C. tropicalis (n = 3).

All strains were subcultured on a selective chromogenic medium for the isolation of yeasts (ChromID®, BioMèrieux) and incubated at 37° for 24 h. A presumptive identification was done by ChromID® and confirmed by Matrix-Assisted Laser Desorption Ionization Time Of Flight Mass Spectrometry (Bruker Daltonics, Bremen, Germany; score > 2).

The trial was carried out at the Laboratory of Microbiology and Virology of the IRCCS Policlinico San Matteo in Pavia. 

The evaluation of the antifungal activity was performed by setting up an agar diffusion method [33,34].

It was not possible to perform a quantitative study using the methods of determination of the Minimum Inhibiting and Fungicidal Concentration (MIC and MFC), due to the insolubility of the ozonized oil in culture broth, with the product being strongly hydrophobic. Not even the addition of a detergent such as Tween 80, as indicated in the study by Sechi et al. [10], could solubilize the gel. 

In order to perform the analysis, aA suspension was prepared by dissolving Candida colonies in distilled water to have a turbidity of 0.5 MacFarland.

ChromID® agar plates were seeded with a buffer soaked with the fungal suspension. Subsequently, two wells were made in each agar plate, one of which was filled with 150 μL of ozonized gel and the other with 150 μL of chlorhexidine.

The plates were then incubated for 48 h at 37 °C. The results were read after 48 h, due to the different growth times of Candida strains. The efficacy of GeliO_3_ and Plak gel® 0,2% was evaluated by measuring the growth inhibition diameter formed around the wells. The experiment was repeated three times for each strain.

Results were recorded in terms of the average diameter of inhibition zone (mm). Data were submitted to statistical analysis using a computer software (R version 3.1.3, R Develop-ment Core Team, R Foundation for Statistical Computing, Wien, Austria). Descriptive statistics were calculated for all the groups tested and reported mean, standard deviation, minimum, median, and maximum values. A *t*-test was applied, comparing the results of the two gels in the different Candida Species tested.

Significance for all statistical tests was predetermined at P < 0.05.

## 3. Results

Results are summarized in Table 1. Both GeliO_3_ and Plak gel® have shown an antifungal activity against all the *Candida* species considered *(C. albicans, C. parapsilosis, C. glabrata, C. tropicalis*). The diameters of mycotic growth inhibition were obtained from the arithmetic average of the inhibition diameters measured in the different tests. The values obtained for GeliO3 vary from a minimum of 15 mm for *C. tropicalis* to a maximum of 19 mm for *C. glabrata*, while, for the Plak gel®, the values obtained are very similar for the different species of *Candida,* varying from 16 to 17 mm. All the *Candida* species considered have been shown to be sensitive to chlorhexidine in equal measures. As for GeliO_3_, *Candida* strains showed a slightly different sensitivity among species: greater for *C. glabrata* and *C. albicans*, lower for *C. parapsilosis* and *C. tropicalis*. No significant differences were reported comparing the two gels (P > 0.05) for all the Candida Species tested. However, these data show that both products have an effective antifungal activity.

## 4. Discussion

The GeliO_3_ antimycotic activity evaluation, using an agar diffusion semi-quantitative method, has provided, as a preliminary result, its efficacy against Candida. Although the insolubility and strong hydrophobicity of GeliO_3_ were a limitation of the in vitro study, they could, however, represent an advantage in the clinical scenery, where just these characteristics allow the gel to remain on the lesion without being immediately solubilized by the salivary flow. A second advantage could be the ability of GeliO_3_ to penetrate the hydrophobic biofilm formed by *Candida*. On the contrary, Plak Gel® tends to have a limited persistence on the mucosa, being a water-based gel. The prolonged permanence of ozone on the lesion offers a great clinical advantage, and indeed, many studies highlighted that the time of exposure of microorganisms to ozone is one of the main variables in the evaluation of antifungal and antibacterial efficacy [35].

Fernandez Torres et al. [35], in a study on the fungicidal power of an ozonized vegetable oil, said that contact time has the greatest influence on killing yeasts. This consideration could raise doubts about any unwanted effects, such as tissue damage that could be caused by antiseptics left in place for a long time. Azarpazhooh et al. [36], in a literature review, stated that there is strong evidence of the biocompatibility of ozone toward epithelial cells, gingival fibroblasts and periodontal cells, supporting an absolute absence of risks for the prolonged use of ozone-based products.

This is in accordance with the observations of Tiwari et al. [37], who reported that the application of ozone has no adverse effects but, on the contrary, can offer benefits to oral tissues, such as the remission of mucosal lesions, and the increase in the turnover of oral epithelial cells with speed wound healing.

Ozone performs its action against microorganisms and not against human cells. Indeed, its germicidal action is based on a transient oxidative stress that is lethal for microorganisms due to their poor antioxidant defenses. Some microorganisms, including mycetes, lack enzymes such as catalase and glutathione peroxidase, which are instead present in human cells. These enzymes make the cellular defense system capable of neutralizing the oxidative action given by ozone [38,39].

The antimycotic effectiveness of GeliO_3_ is due to the oxidation of microorganisms through the slow release of peroxides. Ozone oxidizes phospholipids and lipoproteins, which are part of the fungi cell wall, damaging their integrity so as to infiltrate inside the cell, oxidize glycoproteins and glycolipids, and block the enzymatic function. The combination of these reactions causes the inhibition of growth that occurs mainly in certain phases: proliferating cells are considered the most sensitive [40,41].

## 5. Conclusions

Further studies will be needed to confirm the GeliO_3_ effective clinical efficacy on oral candidiasis. It would be interesting to test the ozonized gel in vivo by monitoring the lesion evolution, and also to investigate possible synergies with first-line drugs.

It may also be interesting to study its activity in comparison to that of traditional antifungal drugs, and especially to assess the sensitivity to ozone by *Candida* strains resistant to polyene and azole drugs. A positive result would make ozone a valid alternative local therapy in the increasingly frequent cases of drug resistance. 

## Figures and Tables

**Table 1:** Growth inhibition halos (mm) of the different groups. No significant differences were observed among groups (P > 0.05).

Code	*Candida* Species	Treatment	Mean	SD	Min	Max
ALB CHX	*Candida albicans*	Plak gel®	16.60	0.97	16.00	19.00
ALB OZO	*Candida albicans*	GeliO_3_	17.40	0.84	16.00	19.00
PAR CHX	*Candida parapsilosis*	Plak gel®	17.40	1.17	15.00	19.00
PAR OZO	*Candida parapsilosis*	GeliO_3_	16.60	0.97	16.00	19.00
GLA CHX	*Candida glabrata*	Plak gel®	17.50	0.71	17.00	19.00
GLA OZO	*Candida glabrata*	GeliO_3_	18.90	1.85	15.00	20.00
TRO CHX	*Candida tropicalis*	Plak gel®	16.90	1.20	16.00	19.00
TRO OZO	*Candida tropicalis*	GeliO_3_	16.10	1.60	15.00	19.00

## References

[1] Hsieh C.W., Huang L.Y., Tschen E.F.T., Chang C.F., Lee C.F. (2010). Five novel anamorphic, ascomycetous yeast species associated with mushrooms and soil. FEMS Yeast Res..

[2] Martins M., Henriques M., Ribeiro A.P., Fernandes R., Goncalves V., Seabra A., Azeredo J., Oliveira R. (2010). Oral *Candida* carriage of patients attending a dental clinic in Braga, Portugal. Rev. Iberoam. Micol..

[3] Scully C., EI-Kabir M., Samaranayake L.P. (1994). *Candida* and Oral Candidosis: A Review. Crit Rev. Oral Biol Med..

[4] Vazquez J.A. (2010). Invasive fungal infections in the intensive care unit. Semin. Respir. Crit. Care. Med..

[5] Rodloff A.C., Koch D., Schaumann R. (2011). Epidemiology and antifungal resistance in invasive candidiasis. Eur. J. Med. Res..

[6] Garcia-Cuesta C., Sarrion-Pérez M.G., Bagán J.V. (2014). Current treatment of oral candidiasis: A literature review. J. Clin. Exp. Dent..

[7] Amin L.E. (2018). Biological assessment of ozone therapy on experimental oral candidiasis in immunosuppressed rats. Biochem. Biophys. Rep..

[8] De Rosa F.G., Garazzino S., Pasero D., Perri G., Ranieri M. (2009). Invasive candidiasis and candidemia: New guidelines. Minerva Anestesiologica.

[9] Ellepola A.N., Samaranayake L.P. (2001). Adjunctive use of chlorhexidine in oral candidoses: A review. Oral Dis.

[10] Sechi L.A., Lezcano I., Nunez N., Espim M., Duprè I., Pinna A., Molicotti P., Fadda G., Zanetti S. (2001). Antibacterial activity of ozonized sunflower oil (Oleozon). J. Appl. Microbiol..

[11] Lezcano I., Nuñez N., Espino M., Gómez M. (2000). Antibacterial activity of ozonized sunflower oil, oleozon, against *Staphylococcus aureus* and *Staphylococcus epidermidis*. Ozone Sci. Eng..

[12] Grootveld M., Baysan A., Siddiqui N., Sim J., Silwood C., Lynch E., Lynch E. (2004). History of the Clinical Applications of Ozone. Ozone: The Revolution in Dentistry.

[13] Bocci V. (2004). Ozone as Janus: This controversial gas can be either toxic or medically useful. Mediators. Inflamm..

[14] Baysan A.L.E., Lynch E. (2004). Antimicrobial Effects of Ozone on Caries. Ozone: The Revolution in Dentistry.

[15] Nogales C.G., Ferrari P.H., Kantorovich E.O., a Lage-Marques J. (2008). Ozone therapy in medicine and dentistry. J. Contemp. Dent. Pract..

[16] Srinivasan S.R., Amaechi B.T. (2019). Ozone: A paradigm Shift in Dental Therapy. J. Glob. Oral Heal..

[17] Smith N.L., Wilson A.L., Gandhi J., Vatsia S., Khan S.A. (2017). Ozone therapy: An overview of pharmacodynamics, current research, and clinical utility. Med. Gas. Res..

[18] Elvis A.M., Ekta J.S. (2011). Ozone therapy: A clinical review. J. Nat. Sci. Biol. Med..

[19] Borges G.Á., Elias S.T., da Silva S., de Oliveira Magalhães P., Macedo S.B., Ribeiro A.P.D., Guerra E.N.S. (2017). In vitro evaluation of wound healing and antimicrobial potential of ozone therapy. J. Cranio-Maxillo. Surg..

[20] Suh Y., Shrey P., Re K., Gandhi J., Joshi G. (2019). Clinical utility of ozone therapy in dental and oral medicine. Med. Gas. Res..

[21] Legeay G., Poncin-Epaillard F., Arciola C.R. (2006). New surfaces with hydrophilic/hydrophobic characteristics in relation to (no)bioadhesion. Int. J. Artif. Organs..

[22] Wang M., Tang T. (2018). Surface treatment strategies to combat implant-related infection from the beginning. J. Orthop Translat..

[23] Pallavicini P., Arciola C.R., Bertoglio F., Curtosi S., Dacarro G., D’Agostino A., Ferrari F., Merli D., Milanese C., Rossi S. (2017). Silver nanoparticles synthesized and coated with pectin: An ideal compromise for anti-bacterial and anti-biofilm action combined with wound-healing properties. J. Colloid Interface Sci..

[24] Gristina A.G., Naylor P., Myrvik Q. (1988). Infections from biomaterials and implants: A race for the surface. Med. Prog Technol..

[25] El Hadary A.A., Yassin H.H., Mekhemer S.T., Holmes J.C., Grootveld M. (2011). Evaluation of the effect of ozonated plant oils on the quality of osseointegration of dental implants under the influence of Cyclosporin A. An in vivo study. J. Oral Implantol..

[26] Yücesoy T., Seker E.D., Cenkcı E., Yay A., Alkan A. (2019). Histologic and Biomechanical Evaluation of Osseointegrated Miniscrew Implants Treated with Ozone Therapy and Photobiomodulation at Different Loading Times. Int. J. Oral. Maxillofac. Implants..

[27] Arciola C.R., Montanaro L., Moroni A., Giordano M., Pizzoferrato A., Donati M.E. (1999). Hydroxyapatite-coated orthopaedic screws as infection resistant materials: In vitro study. Biomaterials.

[28] Predoi D., Iconaru S.L., Predoi M.V., Stan G.E., Buton N. (2019). Synthesis, Characterization, and Antimicrobial Activity of Magnesium-Doped Hydroxyapatite Suspensions. Nanomaterials (Basel)..

[29] Van Dijk I.A., Beker A.F., Jellema W., Nazmi K., Wu G., Wismeijer D., Krawczyk P.M., Bolscher J.G., Veerman E.C., Stap J. (2017). Histatin 1 Enhances Cell Adhesion to Titanium in an Implant Integration *Model*. J. Dent. Res..

[30] Harmankaya N., Igawa K., Stenlund P., Palmquist A., Tengvall P. (2012). Healing of complement activating Ti implants compared with non-activating Ti in rat tibia. Acta Biomater..

[31] Sunarso, Toita R., Tsuru K., Ishikawa K. (2016). A superhydrophilic titanium implant functionalized by ozone gas modulates bone marrow cell and macrophage responses. J. Mater. Sci Mater. Med..

[32] Turner S.A., Butler G. (2014). The Candida pathogenic species complex. Cold Spring Harb Perspect Med..

[33] Poggio C., Arciola C.R., Cepurnykh S., Chiesa M., Scribante A., Selan L., Imbriani M., Visai L. (2012). In vitro antibacterial activity of different self-etch adhesives. Int. J. Artif. Organs..

[34] Poggio C., Colombo M., Scribante A., Sforza D., Bianchi S. (2012). In vitro antibacterial activity of different endodontic irrigants. Dent Traumatol..

[35] Fernández Torres I., Curtiellas Piñol V., Sánchez Urrutia E., Gómez Regueiferos M. (2006). In vitro antimicrobial activity of ozonized theobroma oil against *Candida albicans*. Ozone Sci. Eng..

[36] Azarpazhooh A., Limeback H. (2008). The application of ozone in dentistry: A systematic review of literature. J. Dent..

[37] Tiwari S., Avinash A., Katiyar S., Iyer A.A., Jain S. (2017). Dental applications of ozone therapy: A review of literature. Saudi J. Dent. Res..

[38] Nagayoshi M., Fukuizumi T., Kitamura C., Yano J., Terashita M., Nishihara T. (2004). Efficacy of ozone on survival and permeability of oral microorganisms. Oral Microbiol. Immunol..

[39] Bocci V. (1994). Autohaemotherapy after Treatment of Blood with Ozone. A Reappraisal. J. Int. Med. Res..

[40] Thanomsub B., Anupunpisit V., Chanphetch S., Watcharachaipong T., Poonkhum R., Srisukonth C. (2002). Effects of ozone treatment on cell growth and ultrastructural changes in bacteria. J. Gen. Appl. Microbiol..

[41] Bocci V., Luzzi E., Corradeschi F., Silvestri S. (1994). Studies on the biological effects of ozone: 6. Production of transforming growth factor 1 by human blood after ozone treatment. J. Biol. Regul. Homeost. Agents..

